# An miRNA-mRNA integrative analysis in human placentas and mice: role of the Smad2/miR-155-5p axis in the development of fetal growth restriction

**DOI:** 10.3389/fbioe.2023.1159805

**Published:** 2023-05-18

**Authors:** Jia-Xing Wu, Ming Shi, Bao-Ming Gong, Bao-Wei Ji, Cheng-Chen Hu, Gui-Cheng Wang, Lei Lei, Chao Tang, Ling V. Sun, Xiao-Hui Wu, Xue Wang

**Affiliations:** ^1^ State Key Laboratory of Genetic Engineering and National Center for International Research of Development and Disease, Collaborative Innovation Center of Genetics and Development, Institute of Developmental Biology and Molecular Medicine, School of Life Sciences, Fudan University, Shanghai, China; ^2^ Department of Obstetrics and Gynecology, Xin Hua Hospital Affiliated to Shanghai Jiao Tong University School of Medicine, Shanghai, China; ^3^ School of Microelectronics, SINO-SWISS Institute of Advanced Technology, Shanghai University, Shanghai, China; ^4^ Dongguan Key Laboratory of Medical Bioactive Molecular Developmental and Translational Research, Guangdong Provincial Key Laboratory of Medical Molecular Diagnostics, Guangdong Medical University, Dongguan, China; ^5^ Department of Nephrology, Children’s Hospital of Fudan University, Shanghai, China; ^6^ Department of Obstetrics and Gynecology, East Hospital, School of Medicine, Tongji University, Shanghai, China; ^7^ National Clinical Research Center for Child Health, The Children’s Hospital, Zhejiang University School of Medicine, Hangzhou, China

**Keywords:** fetal growth restriction age, placenta, MiRNA-mRNA integrative analysis, Smad2/miR-155-5p axis, villous trophoblast cells

## Abstract

**Introduction:** Functional disorder of the placenta is the principal cause of fetal growth restriction (FGR), usually cured with suitable clinical treatment and good nursing. However, some FGR mothers still give birth to small for gestational age (SGA) babies after treatment. The ineffectiveness of treatment in such a group of patients confused physicians of obstetrics and gynecology.

**Methods:** In this study, we performed a microRNA-messenger RNA integrative analysis of gene expression profiles obtained from Gene Expression Omnibus. Differentially expressed genes were screened and checked using quantitative polymerase chain reaction. Target genes of significantly changed microRNA were screened and enriched for Gene Ontology and Kyoto Encyclopedia of Genes and Genomes pathway analyses. Function of the obtained microRNA-messenger RNA was evaluated using HTR-8/SVneo trophoblast cells, human umbilical vein endothelial cells, and heterozygote male mice.

**Result:** MiR-155-5p was upregulated (*p* = 0.001, fold-change = 2.275) in fetal-side placentals. Among the hub genes identified as key targets for miR-155-5p in fetal reprogramming, Smad2 was downregulated (*p* = 0.002, fold change = 0.426) and negatively correlated with miR-155-5p expression levels (r = −0.471, *p* < 1.0 E – 04) in fetal-side placental tissues. The miR-155-5p mimic blocks Smad2 expression and suppresses villous trophoblast cell and endothelial cell function (proliferation, migration, and invasion), indicating a close relationship with placental development. Luciferase assays further confirmed the targeting of miR-155-5p to Smad2. Furthermore, Smad2^+/−^ heterozygote male mice were born small with low body weight (*p* = 0.0281) and fat composition (*p* = 0.013) in the fourth week post-natal.

**Discussion:** We provide the first evidence of the role of the Smad2/miR-155-5p axis in the placental pathologies of FGR. Our findings elucidate the pathogenesis of FGR and provide new therapeutic targets.

## 1 Introduction

Fetal growth restriction (FGR) is a pregnancy complication describing a fetus that does not grow to its full potential due to pathological compromise. FGR fetuses could develop small for gestational age (SGA), which is small and at less than the 10th percentile in weight for healthy fetuses and more prone to disease and death. Globally, 5%∼10% of all births are FGR (Froen et al., 2004), leading to perinatal mortality and being responsible for 30% of stillborn infants (Nardozza et al., 2017). FGR newborns are more likely to experience learning difficulties, cognitive problems, and attention problems than their appropriately grown counterparts (O'Keeffe et al., 2003; Heinonen et al., 2010; Sharma et al., 2016). Although they could catch-up growth in early life, FGR patients are more likely to be vulnerable to developing metabolic diseases such as insulin resistance, cardiovascular disease, diabetes mellitus, or adult-onset non-communicable diseases (Clayton et al., 2007; Gluckman et al., 2009; Lawn et al., 2014). Except for genetic factors, numerous FGR babies develop pathological changes in placentas. Multiple placental phenotypes of FGR caused by different pathologies exist. Early-onset FGR, typically diagnosed based on a finding of a small-for-gestational-age (SGA) fetus with abnormal blood flow in the umbilical artery recorded by Doppler ultrasound, is generally caused by impaired extravillous trophoblast (EVT) invasion and spiral artery remodeling (Pollheimer et al., 2018). Late-onset FGR (≥32 weeks) may occur in the lacking of suboptimal EVT invasion and uterine vascular remodeling. Instead, such patients could be characterized by a small size of the placenta, abnormal villous trophoblast turnover and syncytiotrophoblast shedding, a reduced density of placental blood vessels, oxidative or nitrative stress, reduced expression and activity of nutrient transporters, and/or evidence of placental inflammation (Araujo Junior et al., 2021). However, its exact etiology remains unclear. EVT cell invasion is orchestrated by a complex array of regulatory mechanisms and is controlled by various gene regulatory and signaling networks. Some FGR patients without other illnesses still give birth to SGA fetuses (gestational age >37 weeks) after clinical treatment and good clinical care, making failed therapy and confusing clinicians.

MicroRNAs (miRNAs), small single-stranded non-coding RNA discovered in various organisms (Enright et al., 2003; Ambros, 2004; Voinnet, 2009), are essential regulators of epigenetic inheritance at transcriptional and post-transcriptional levels (Ha and Kim, 2014). Some miRNAs carried by peripheral blood or external vesicles are credible biomarkers of certain diseases (van de Vrie et al., 2017; Ma, 2018), while other distinct miRNA contributors to pathological progression are considered therapeutic targets (Rupaimoole and Slack, 2017). Moreover, miRNAs are closely associated with uterine circumstances, placental pathology, and epigenetic features of offspring. For example, miR-210 was consistently dysregulated during pregnancy in women with preeclampsia (Bounds et al., 2017), miR-145-5p and miR-875-5p were identified as biomarkers of gestational diabetes mellitus (Zamanian Azodi et al., 2019), and circulating miR-323-3p could add substantial diagnostic accuracy to a panel including human chorionic gonadotropin and progesterone for the diagnosis of ectopic pregnancy (Zhao et al., 2012). Hence, studies that shed light on the regulation of miRNAs in EVT cell invasion are helpful for understanding the basic pathogenesis of FGR. In addition, previously reported miRNAs for FGR have included patients with multiple clinical diseases (Maccani et al., 2011; Ostling et al., 2019), and research on simple FGR is urgently needed.

In this study, we focused on post-transcriptional regulation in trophoblastic cells via transcriptome-level analysis of placental tissues. Publicly available miRNA and RNA profiles from Gene Expression Omnibus (GEO) repository were assigned to an FGR or a healthy group; differentially expressed genes (DEGs) between the two groups were determined and further verified on placental tissues using quantitative polymerase chain reaction (qPCR) and immunohistochemistry (IHC). We found that miR-155-5p was significantly highly expressed gene in FGR placentas and regulated the proliferation and invasion of trophoblast cells. Additionally, as the target gene of miR-155-5p, Smad2 was downregulated in the EVT cells and fetal-side placental tissues. Our novel findings revealed post-translational regulations of miRNA on the pathogenesis of placentas with simple type FGR, which offers insights into pregnancy complications and fetal development.

## 2 Materials and methods

### 2.1 Data collection, processing, and differentially expressed gene screening

We searched “FGR”, “tissue” and “*Homo sapiens*” in the GEO repository. A dataset GSE93174 of miRNA profiles for placentas was downloaded. Message RNA profiles of placentas in six datasets (GSE98224, GSE75010, GSE100415, GSE24129, GSE10588, and GSE30186) were downloaded from the GEO repository and integrated analyzed as previously described (Wang et al., 2020). To reduce other pathogens, we excluded gene profiles containing information on “preeclampsia (PE),” “large for gestational age (LGA),” and “preterm.” The selected data were collected as a cohort and assigned to the FGR or healthy group for subsequent analysis. The clinical characteristics of the cohort are listed in Supplementary Materials (GSE_profile_information.xls) and were summarized (Supplementary Tables S1, S2). We used WebPlotDigitizer (https://automeris.io/WebPlotDigitizer) online software to obtain miR-155-5p relative expression data of placentas in other literature (Hromadnikova et al., 2022).

The limma package (https://bioconductor.org/packages/release/bioc/html/limma.html) in R was used to screen differentially expressed genes (DEGs) for raw or normalized data. Background correction and quantile normalization of raw data were performed using the ComBat model. Differentially expressed miRNAs (DEMs) were generated, and significant DEMs were screened with thresholds *p* < 0.05, and |log2 (fold change)| >0.3. Volcano plots and heatmaps were drawn using R language.

### 2.2 Bioinformatics analysis

Micro RNA data were searched using miRBase (http://www.mirbase.org/). miRWalk (http://mirwalk.umm.uni-heidelberg.de/; accessed on 22 June 2021) was used to access databases (TargetScan, PicTar, RNA22, PITA, miRanda, and miRDB) to predict target genes of miRNA. Target genes predicted that at least four databases were regarded as candidates. A Venn diagram was generated using R.

A Kyoto Encyclopedia of Genes and Genomes (KEGG) pathway enrichment analysis of candidates was performed to discover pathways that were regulated by miR-155-5p using the “GeneAnswers” package (http://www.bioconductor.org/packages/release/bioc/html/GeneAnswers.html). A Gene Ontology (GO) enrichment analysis was performed using the “GOstats” package (http://www.bioconductor.org/packages/release/bioc/html/GOstats.html) to evaluate the functional and biological significance of miR-155-5p. Protein–protein interaction (PPI) network information for predicted target genes was obtained using the Search Tool for the Retrieval of Interacting Genes (STRING, http://www.string-db.org/). Genes with at least five branches in networks were identified as key genes.

### 2.3 Study population

Twenty-three FGR patients (without other complications) and 40 healthy pregnant women were recruited from January 2019 to December 2021 at the Xin Hua Hospital Affiliated to Shanghai Jiao Tong University School of Medicine, Shanghai, China. This study was approved by the ethics committee of the hospital (approval number: XHEC-D-2022-132). All recruited patients provided signed informed consent prior to placental tissue collection. Initial recruitment for participants was performed on pregnant women at a gestational age of approximately 28 weeks, and cases of FGR were primarily screened using fetoplacental Doppler. With the progression of pregnancy, some cases were relieved. Finally, only newborns with SGA at delivery belonged to the FGR group and donated placental tissues. Donors met the following criteria: 1) 20–35 years of age and gestational age ≥37 weeks; 2) singleton pregnancy; 3) newborns with a birth weight 10 percent lower than that of a normal baby at the same gestational age; 4) non-drinking and non-smoking; and 5) no other pregnancy complications or genetic deficiency currently or previously.

### 2.4 Sample processing

Tissue samples were retrieved from placentas within 30 min after delivery, frozen in liquid nitrogen, and subsequently stored at −80°C for RNA extraction or fixed in formalin instantaneously for hematoxylin-eosin (HE) staining and immunohistochemistry (IHC). To avoid spatial discrepancies, small pieces of tissue were cut from six different regions of the placenta. Additionally, each piece was 4–6 cm from the terminal of the umbilical cord and cut into two parts (maternal and fetal sides). Total RNA and miRNA were isolated using an RNeasy Plus Universal Kit (Cat. No. 74134; Qiagen, Hilden, Germany) and miRcute miRNA Isolation Kit (DP501; Tiangen, Beijing, China), respectively, according to the manufacturer’s instructions. RNA quality and quantity were assessed using an electrophoresis device (Tanon 3500; Tanon, Shanghai, China) and NanoDrop™ One Microvolume UV-Vis Spectrophotometer (ND-ONEC-W; Thermo Fisher Scientific, United States), respectively.

### 2.5 Quantitative polymerase chain reaction (qPCR)

Analysis was performed following the Minimum Information for Publication of Quantitative Real-Time PCR Experiment guidelines as previously described [23]. Briefly, isolated RNA was reverse-transcribed using a PrimeScript™ RT Reagent Kit (RR047A; Takara, Kusatsu, Japan), and miRNA was reverse-transcribed using a miRcute Plus miRNA First-Strand cDNA Kit (KR211; Tiangen, Beijing, China). Target gene and miRNA expression levels were detected using a real-time PCR instrument (LightCycler480 II; Roche, Switzerland) and SYBRGreen PCR Kits (FP205 and FP411, respectively; Tiangen, Beijing, China). The relative expression levels of candidate genes were normalized to the reference gene (GAPDH or U6) and calculated using the comparative CT (2^−ΔΔCT^) method. The primer sequences used were GAPDH: (Fw) 5′-GAC​TCA​TGA​CCA​CGT​CCA​TGC-3′ and (Rv) 5′-AGA​GGC​AGG​GAT​GAT​GTT​CTG-3′; Smad2: (Fw) 5′-GTG​TCA​CCA​TAC​CAA​GCA​CTT​GC-3′ and (Rv) 5′-GAC​TCA​AAG​CGA​CAG​ATA​ACA​CG-3′. miRNA primers were purchased from Tiangen (Beijing, China) used as commercial products. Amplification efficiencies of genes ranged from 90% to 115%, and melting curves of tested genes indicated that the amplification was specific.

### 2.6 Hematoxylin-eosin staining and immunohistochemistry of placental tissues

Placentas and mice embryos maintained in formalin were paraffin embedded and sliced for HE staining and histological grading. IHC was also performed to determine the translational level of the target gene. Briefly, after blocking with 10% non-immune serum, sections were incubated with an antibody against Smad2 (12570-1-AP; Proteintech, Chicago, IL, United States; 1.1 μg/mL) at room temperature for 2 h, followed by incubation with the secondary antibody (Santa Cruz Biotechnology, United States) for 1 h. Following incubation in a streptavidin–peroxidase conjugate (Sigma-aldrich, German), the antibody complexes were visualized using diaminobenzidine tetrahydrochloride chromogen for 10 s. Stained tissue sections were observed using an optical microscope (Olympus™ CK 30; Keyens Tokyo, Japan).

### 2.7 Cell culture, transfection, and assays

HTR8/SVneo (HTR8) cells, a human-derived trophoblast cell line, were maintained in DMEM high-glucose medium (10-013-CV; Corning, Manassas, VA, United States) supplemented with 10% fetal bovine serum (Biowest, Nuaillé, France) and 1% penicillin-streptomycin (Gibco, California, United States). Cells were cultured on adherent tissue culture dishes (Thermo Fisher Scientific, Waltham, MA, United States) at 37°C in a 5% CO_2_ incubator. Cells were transfected with miR-155-5p mimic (miR10000646-1-5; RiboBio, Guangzhou, China) or the negative control (miR01201-1-5; RiboBio) using the riboFECTTM CP Transfection Kit (C10511-05; RiboBio) at a density of 50%, and each sample was triplicated (N = 3). Cell viability was tested using the Cell Counting Kit-8 (CCK-8; CK04; Dojindo, Kumamoto, Japan) with a microplate absorbance reader (Gene 5; Epoch Biotech, United States) at 450 nm. Cell migration was assessed using the transwell-24 system (3422, 8 μm; Corning, New York, United States). Briefly, 8 × 10^4^ post-transfected cells were separated into the upper chambers with 150 μL serum-free medium and then plated on the lower chambers with 550 μL culture medium. For cell invasion, membranes of the upper chambers were pre-coated with Matrigel (356,234; Corning, New York, United States). The chambers were incubated for 36 and 48 h for migration and invasion, respectively. Transferred cells found outside the upper chambers were washed gently, fixed, stained, and photographed. Trials were performed in triplicate (N = 3), and stained cells were eluted using 33% cold acetate, and the absorbance was subsequently monitored at 590 nm. Human umbilical vein endothelial cells (HUVEC) were cultured at 37°C in a 5% CO_2_ incubator in Ham’s F-12K medium (N3520; Sigma, United States) supplemented with 100 μg/mL heparin (H3149; Sigma, United States), 50 μg/mL endothelial cell growth supplement (354,006; Corning), 10% fetal bovine serum (Biowest) and 1% penicillin-streptomycin (Gibco). Cell viability and transwell assay were performed using CCK-8 as previously described.

### 2.8 Western blot analysis

Cells were cultured and collected 48 h post-transfection for the Western blot assay. Briefly, the cells were washed twice with cold phosphate-buffered saline and lysed in radioimmunoprecipitation assay buffer (Beyotime Biotechnology, Shanghai, China) on ice. Protein concentration in the supernatant was quantified using a bicinchoninic acid kit (P0012S; Beyotime Biotechnology). Twenty micrograms of protein from each sample were separated using 10% sodium dodecyl sulfate–polyacrylamide gel electrophoresis and transferred to polyvinylidene difluoride membranes (Roche, Basel, Switzerland). After blocking in 5% skim milk/TBS–Tween-20 (1%) and incubation with primary antibodies against Smad2 (12570-1-AP; Proteintech; 0.22 μg/mL) and GAPDH (10494-1-AP; Proteintech; 0.0165 μg/mL), membranes were incubated with secondary antibodies and visualized using a chemiluminescence-based detection system (Pierce Biotechnology, Rockford, IL, United States). The intensity of protein spots was analyzed using densitometry and normalized to GAPDH expression. Blotings of target bands were evaluated by ImageJ software for grayscale levels.

### 2.9 Luciferase reporter gene assay

Luciferase assays were performed using the Dual-Luciferase Reporter Assay System (E1910; Promega, United States). The wild-type (WT) and mutated 3′UTR of Smad2 were amplified from complementary DNA of HTR8 cells and subsequently cloned into the pmir-GLO reporter vector. For luciferase reporter assays, miR-155-5p mimics and negative controls were co-transfected with WT or mutated 3′UTRs into HEK293T cells.

### 2.10 Animals and metabolic assays

FVB mice were housed under a 12/12 h light/dark cycle with free access to water and food. All mutants (Muts) were generated on an FVB/NJ background. We generated Smad2^+/−^ heterozygote mice by disrupting Smad2 expression via a piggyBac(PB) insertion according to PBmice techniques of our institute (Sun et al., 2008). Mapping information on PB insertions in Smad2 can be found in the PBmice database (idm.fudan.edu.cn/PBmice). All animal experiments were performed in accordance with the guidelines of the Institutional Animal Care and Use Committee of the Institute of Developmental Biology and Molecular Medicine. The quantity and ratio of body fat and the weight of mice (4 weeks after birth, *n* = 7) were measured using a miniSpec NMR instrument (Bruker, Germany). Mice (*n* = 3) were injected intraperitoneally with miR-155-5p agomiR (miR40000165-4-5, RiboBio) at a dose of 0.1 nmol/g with in 0.1 mL saline for 3 days from E13.5. A scramble agomiR (miR4N0000001-4-5, RiboBio) was used as negative control. Placentas and embryos were collected for morphology observation and IHC assay at E18.5.

### 2.11 Statistical analyses

Group differences were analyzed using Student’s t-test or one-way analysis of variance (*p < 0.05; **p < 0.005). SEM represents the variation between sample means. A Pearson correlation assay was applied to qPCR data to obtain correlation coefficients for the relationships between the expression levels of miRNAs and those of the target genes.

## 3 Results

### 3.1 Identification of DEMs in SGA-related GEO profiles

**FIGURE 1:**
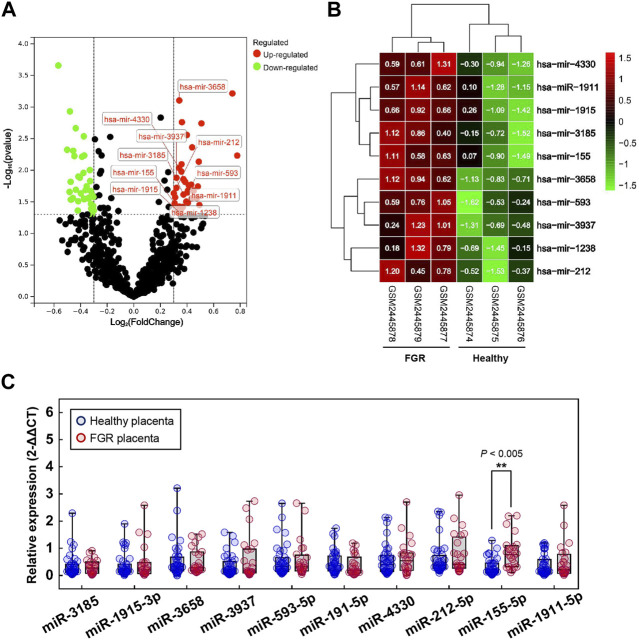
Significant differentially expressed microRNAs (DEMs). **(A)** Volcano map for DEMs; red dots and green dots indicate genes that were upregulated and downregulated (FDR <0.05) in the fetal growth restriction (FGR) group, respectively. The labeled dots indicate significant DEMs with high expression levels (Top 10). **(B)** Two-way hierarchical clustering map of labeled significant DEMs for the healthy group and the FGR group. **(C)** The qPCR analysis of placental tissues to verify the transcript levels of significant DEMs. **p < 0.005.

Profiles downloaded from the GEO dataset were divided into a healthy group and an FGR group after standard normalization. To identify specific miRNAs in placentas that contributed to the development of FGR, 79 DEMs (*p* < 0.05, Log2|fold change| >0.3) were screened and are listed in the Supplementary Materials (DEMs.xls). Ten significantly upregulated DEMs with the highest expression levels were considered candidate biomarkers responsible for pathological features and functional disruptions of FGR placentas (Table 1). A heat map (Figure 1A) and a volcano plot (Figure 1B) were generated for the candidate DEMs. The heat map was distinctly clustered into an FGR group and a healthy group, indicating the relevance of these upregulated genes to FGR. To identify whether these miRNAs are truly upregulated in the placentas of FGR, we performed a qPCR assay on 40 healthy placentas and 23 FGR placental tissues for further verification. miR-155-5p (*p* = 0.001 < 0.005, fold change = 2.275) was successfully verified (Figure 1C). The clinical characteristics of patients involved in qPCR assays are summarized (Table 2). Data mined from the literature also showed upregulated miR-155-5p in FGR and SGA placentas (Supplementary Figure S1), supporting our selection of miR-155-5p.

**TABLE 1:** Significant upregulated differentially expressed microRNAs (DEMs) in placental tissues of fetal growth restriction (FGR) patients.

Gene name	*p*-value	Average expression level	Log (fold change)	Mature sequences of miRNA
hsa-mir-3185	0.024	4.120	0.374	5′-AGA​AGA​AGG​CGG​UCG​GUC​UGC​GG-3′
hsa-miR-1915-5p	0.045	3.663	0.337	5′- ACC​UUG​CCU​UGC​UGC​CCG​GGC​C-3′
hsa-mir-3658	0.001	3.644	0.342	5′- UUU​AAG​AAA​ACA​CCA​UGG​AGA​U-3′
hsa-mir-3937	0.013	3.535	0.321	5′-ACA​GGC​GGC​UGU​AGC​AAU​GGG​GG-3′
hsa-miR-593-5p	0.023	3.412	0.403	5′-AGG​CAC​CAG​CCA​GGC​AUU​GCU​CAG​C-3′
hsa-miR-1238-5p	0.038	3.360	0.369	5′-GUG​AGU​GGG​AGC​CCC​AGU​GUG​UG-3′
hsa-mir-155-5p	0.033	3.253	0.307	5′- UUA​AUG​CUA​AUC​GUG​AUA​GGG​GU-3′
hsa-mir-4330	0.010	3.189	0.358	5′- CCU​CAG​AUC​AGA​GCC​UUG​C-3′
hsa-miR-212-5p	0.019	3.148	0.320	5′-ACC​UUG​GCU​CUA​GAC​UGC​UUA​CU-3′
hsa-miR-1911-5p	0.031	3.067	0.395	5′-UGA​GUA​CCG​CCA​UGU​CUG​UUG​GG-3′

**TABLE 2:** Clinical characteristics of healthy individuals and fetal growth restriction (FGR) patients^.^

Variables	Healthy (*n* = 40)	FGR (*n* = 23)
Age	29.13 ± 3.39	30.30 ± 3.07
Gestational age (weeks)	39.36 ± 0.97	38.64 ± 1.44
Maternal ethnicity	Chinese	Chinese
Tissue type	Placenta	Placenta
Previous pregnancy	1.45 ± 0.71	1.48 ± 0.85
Previous delivery	1.15 ± 0.36	1.09 ± 0.29
Previous FGR	No	No
Previous miscarriage	No	No
Previous hypertensive pregnancy	No	No
Mode proteinuria	No	No
Hellp diagnosis	No	No
FGR diagnosis	No	Yes
Chorioamnionitis diagnosis	No	No
Infant sex (Male/Female)	22/18	11/12
Infant weight (g)	3340.43 ± 334.49	2235.22 ± 223.04^a^
Aparg-score 1 min	9.55 ± 0.50	9.48 ± 0.51

^a^ *p* < 0.01.

HELLP: hemolysis, elevated liver enzymes and low platelets.

### 3.2 Functional enrichment analysis of the miR-155-5p

**FIGURE 2:**
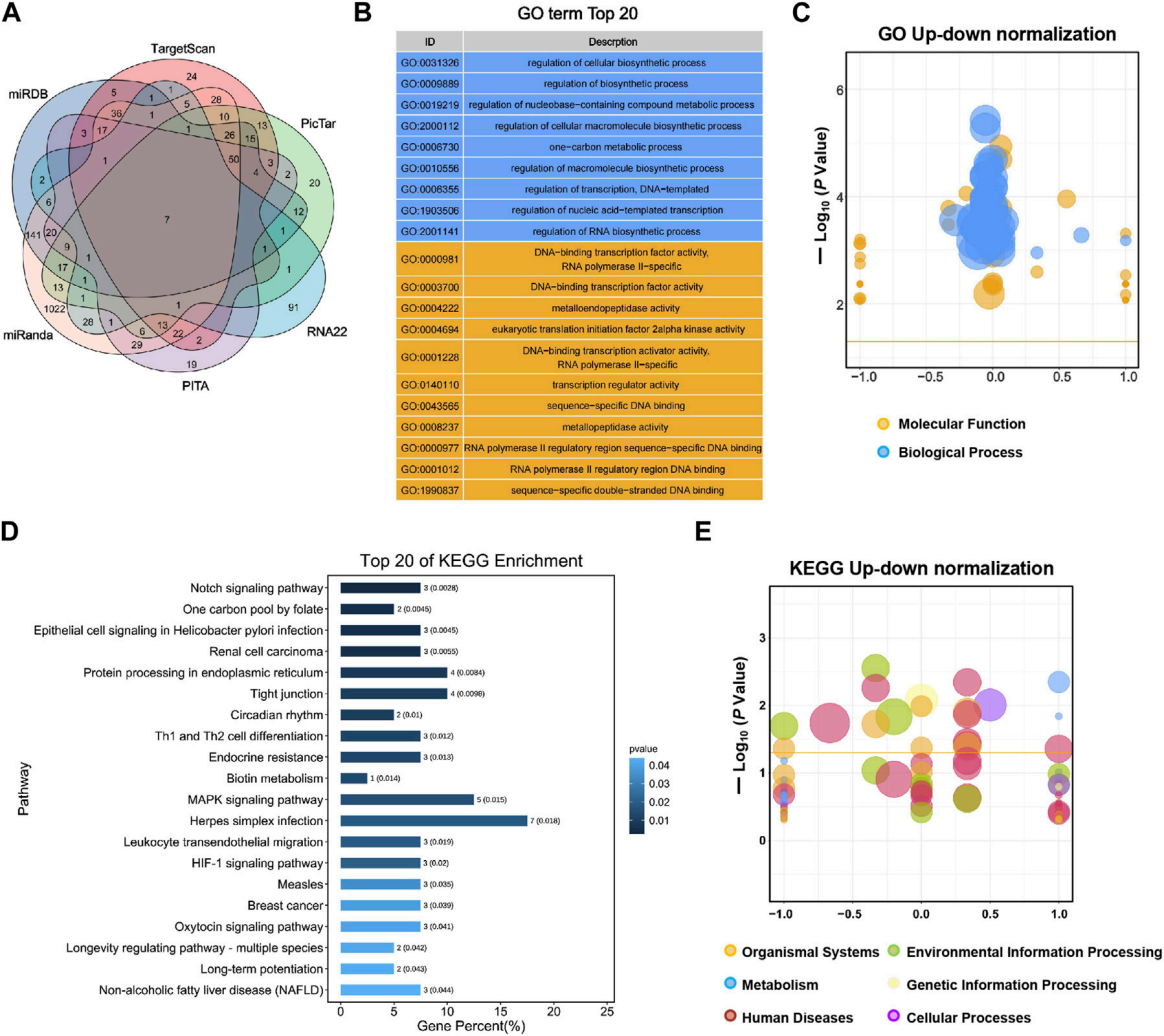
GO enrichment and KEGG analyses for predicted target genes of miR-155-5p. **(A)** Venn diagram with six gene sets predicted from different databases, 128 target genes gathered in at least four sets were adopted for subsequent analyses. **(B)** Top 20 enriched GO terms of target genes. **(C)** Up-down normalization map for top GO terms. **(D)** Top 20 KEGG pathways of predicted target genes. **(E)** Up-down normalization map for top KEGG pathways.

TargetScan, PicTar, RNA22, Miranda, PITA, and miRDB databases were used to predict miR-155-5p targets. The predicted genes are listed in Supplementary Materials (PreditedTargets.xls). A Venn diagram with six gene sets from different databases was generated (Figure 2A). To better understand the cellular functions and biological pathways in which miR-155-5p is involved, 128 target genes gathered in at least four sets in the Venn diagram were adopted for KEGG analyses and GO enrichment. The top 10 enriched GO terms for biological processes, molecular functions, and cellular components are summarized in Table 3. However, as shown in Figures 2B, C, the significantly enriched GO terms were mostly focused on biological processes (e.g., regulation of cellular biosynthetic processes) and molecular functions (e.g., DNA-binding transcription factor activity), indicating a close connection between miR-155-5p and embryonic development. The top enriched KEGG pathways are summarized in Figures 2D, E; Table 4. These 128 target genes were primarily involved in pathways of environmental information processing, such as Notch signaling, MAPK signaling, and HIF-1 signaling, which have been widely reported as pathways closely associated with the development of FGR.

**TABLE 3:** Top 10 enriched significant GO terms of different ontologies for target genes of miR-155-5p.

Ontology	GO id	Description	*p*-value	Gene number
Biological process	0031326	regulation of cellular biosynthetic process	3.77E-06	37
	0009889	regulation of biosynthetic process	5.29E-06	37
	0019219	regulation of nucleobase-containing compound metabolic process	2.04E-05	35
	2000112	regulation of cellular macromolecule biosynthetic process	2.36E-05	34
	0006730	one-carbon metabolic process	2.40E-05	4
	0010556	regulation of macromolecule biosynthetic process	2.84E-05	34
	0006355	regulation of transcription, DNA-templated	4.07E-05	31
	1903506	regulation of nucleic acid-templated transcription	4.11E-05	31
	2001141	regulation of RNA biosynthetic process	4.35E-05	31
	0034654	nucleobase-containing compound biosynthetic process	4.45E-05	35
Cellular component	0043231	intracellular membrane-bounded organelle	5.35E-03	58
	0090575	RNA polymerase II transcription factor complex	6.28E-03	4
	0043227	membrane-bounded organelle	6.99E-03	63
	0036488	CHOP-C/EBP complex	8.27E-03	1
	1990617	CHOP-ATF4 complex	8.27E-03	1
	1990622	CHOP-ATF3 complex	8.27E-03	1
	0044798	nuclear transcription factor complex	8.47E-03	4
	0098636	protein complex involved in cell adhesion	1.03E-02	2
	0005634	nucleus	1.05E-02	42
	0008247	1-alkyl-2-acetylglycerophosphocholine esterase complex	1.24E-02	1
Molecular function	0000981	DNA-binding transcription factor activity, RNA polymerase II-specific	1.16E-05	18
	0003700	DNA-binding transcription factor activity	2.04E-05	18
	0004222	metalloendopeptidase activity	8.71E-05	5
	0004694	eukaryotic translation initiation factor 2alpha kinase activity	1.06E-04	2
	0001228	DNA-binding transcription activator activity, RNA polymerase II-specific	1.10E-04	9
	0140110	transcription regulator activity	1.35E-04	20
	0043565	sequence-specific DNA binding	1.64E-04	18
	0008237	metallopeptidase activity	1.64E-04	6
	0000977	RNA polymerase II regulatory region sequence-specific DNA binding	2.19E-04	16
	0001012	RNA polymerase II regulatory region DNA binding	2.19E-04	16

**TABLE 4:** Twenty significant KEGG pathways for target genes of miR-155-5p.

KEGG id	Pathway	Class	*p*-value	Gene number
ko04330	Notch signaling pathway	Environmental Information Processing	2.79E-03	3
ko00670	One carbon pool by folate	Metabolism	4.48E-03	2
ko05120	Epithelial cell signaling in *Helicobacter pylori* infection	Human Diseases	4.52E-03	3
ko05211	Renal cell carcinoma	Human Diseases	5.49E-03	3
ko04141	Protein processing in endoplasmic reticulum	Genetic Information Processing	8.35E-03	4
ko04530	Tight junction	Cellular Processes	9.80E-03	4
ko04710	Circadian rhythm	Organismal Systems	1.02E-02	2
ko04658	Th1 and Th2 cell differentiation	Organismal Systems	1.24E-02	3
ko01522	Endocrine resistance	Human Diseases	1.34E-02	3
ko00780	Biotin metabolism	Metabolism	1.44E-02	1
ko04010	MAPK signaling pathway	Environmental Information Processing	1.46E-02	5
ko05168	Herpes simplex infection	Human Diseases	1.81E-02	7
ko04670	Leukocyte transendothelial migration	Organismal Systems	1.88E-02	3
ko04066	HIF-1 signaling pathway	Environmental Information Processing	2.01E-02	3
ko05162	Measles	Human Diseases	3.50E-02	3
ko05224	Breast cancer	Human Diseases	3.86E-02	3
ko04921	Oxytocin signaling pathway	Organismal Systems	4.05E-02	3
ko04213	Longevity regulating pathway - multiple species	Organismal Systems	4.21E-02	2
ko04720	Long-term potentiation	Organismal Systems	4.33E-02	2
ko04932	Non-alcoholic fatty liver disease (NAFLD)	Human Diseases	4.38E-02	3

### 3.3 MiR-155-5p decreased the proliferation, migration, and invasion abilities of HTR8/SVneo cells

**FIGURE 3:**
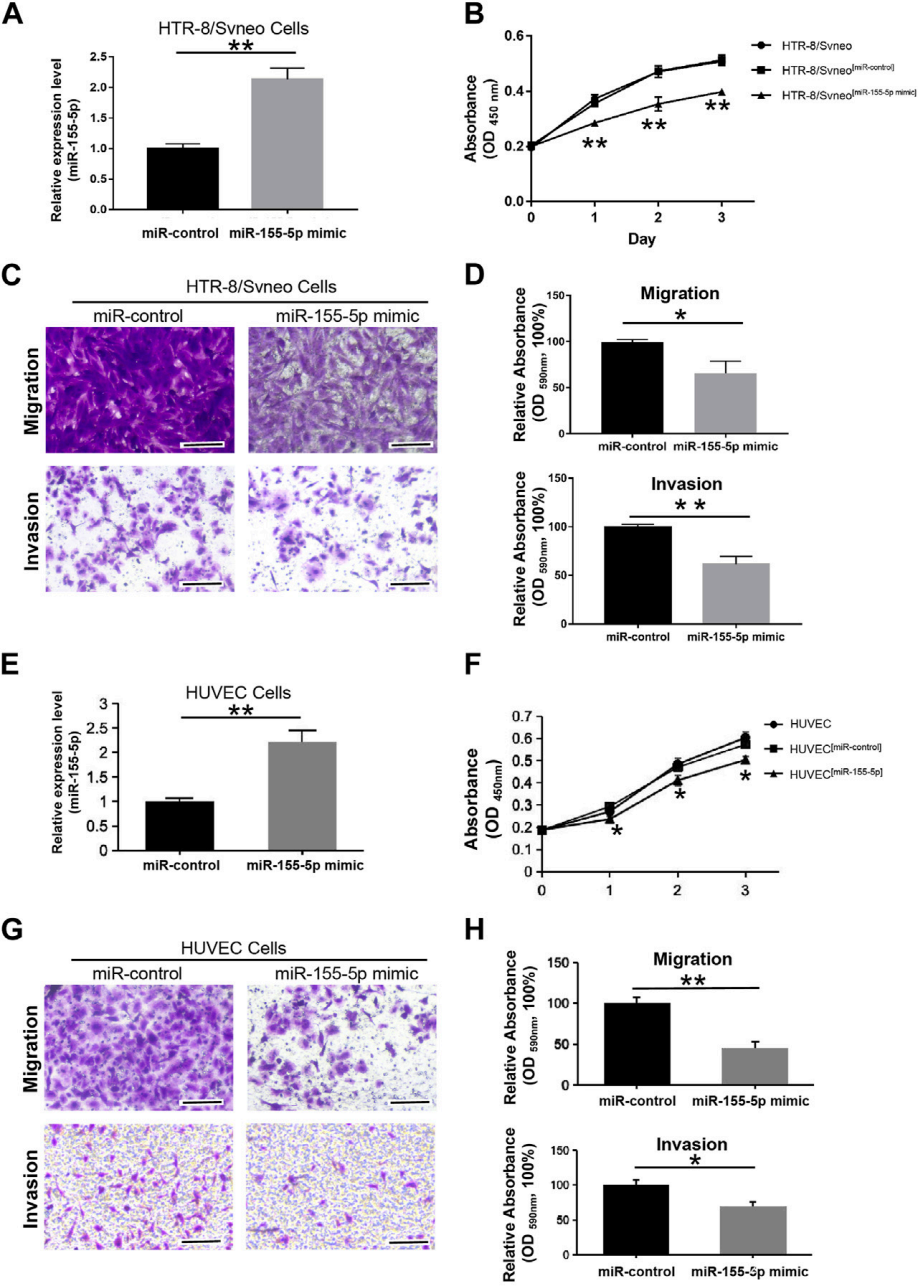
MiR-155-5p decreased the proliferation, migration, and invasion abilities of HTR8/SVneo and HUVEC cells. **(A)** The significant upregulation of miR-155-5p in HTR8/SVneo cells was verified by a qPCR assay. **(B)** CCK-8 assay of HTR8/SVneo cells at 24, 48, and 72 h post-transfection. **(C)** Transwell assays of HTR8 cells post-transfection. **(D)** Quantitative HTR-8/SVneo cells abilities of migration and invasion post-transfection by measuring acetic acid elution buffer. **(E)** The significant upregulation of miR-155-5p in HUVEC cells was verified by a qPCR assay. **(F)** CCK-8 assay of HUVEC cells. **(G)** Transwell assays of HUVEC cells post-transfection. **(H)** Quantitative HUVEC cells abilities of migration and invasion. The bars represent 100 μm *p < 0.05, **p < 0.005.

To explore the regulatory mechanism of miR-155-5p in HTR8 cells, we first evaluated cell proliferation when miR-155-5p upregulated. At 48 h post-transfection with the miR-155-5p mimic, the translational level of miR-155-5p in cells was confirmed to be significantly upregulated (Figure 3A). The CCK-8 assay indicated that cell proliferation significantly decreased at 24, 48, and 72 h post-transfection (Figure 3B). To investigate the potential role of miR-155-5p in placental development and villous trophoblast cell function, transwell assays were performed on HTR8 cells post-transfection. Consequently, the migration and invasion abilities of HTR-8/SVneo cells were significantly reduced (Figures 3C, D), further suggesting a correlation between upregulated miR-155-5p and disruption of placentas during FGR development. The HUVEC cells have shown similar results (Figures 3E–H).

### 3.4 Expression level of Smad2 is negatively correlated to that of miR-155-5p in fetal-side SGA placentas

**FIGURE 4:**
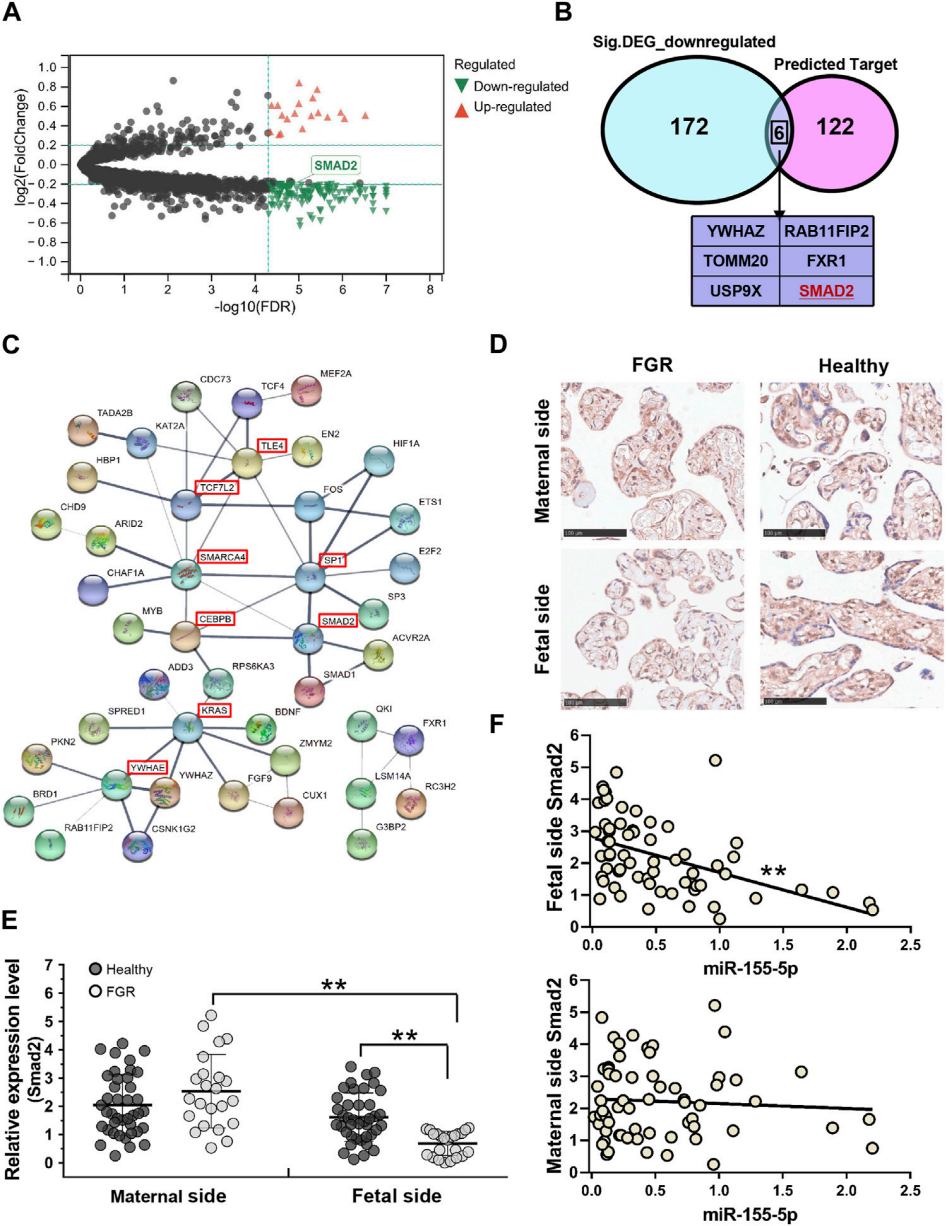
Smad2 significant downregulated in fetal-side FGR placentas. **(A)** Volcano map for DEGs; red dots and green dots indicate genes that were upregulated and downregulated in the FGR group, respectively. **(B)** Venn diagram of 128 predicted target genes of miR-155-5p and 178 green dots indicated genes in **(A)**; six genes owning to the intersection. **(C)** Predicted target genes of miR-155-5p were used to generate a PPI network using the STRING online database; hub genes are indicated in red boxes. **(D)** Immunohistochemistry results for placenta tissues to confirm Smad2 protein levels. Scale bars indicate 100 μm. **(E)** The qPCR analysis of placental tissues to determine the transcript levels of Smad2. **(F)** The expression level of Smad2 was significantly associated with miR-155-5p in fetal-side placenta tissues. *p < 0.05, **p < 0.005.

To identify mRNAs associated with FGR development and miR-155-5p, 96 GSM profiles from six GEO datasets were downloaded, screened, and divided into a healthy group (68 healthy subjects) and an FGR group (28 FGR subjects). A total of 178 downregulated DEGs (*p* < 5.0 E – 07, Log2|fold change| < – 0.2) were screened (Figure 4A, green dots) and are listed in the Supplementary Materials (Down_DEGs.xls). Smad2 belongs to the intersection of Figure 4B, indicating it is significantly downregulated in FGR placentas and predicted as a target of miR-155-5p. A PPI network was generated using 128 predicted target genes, Smad 2 was also defined as a key protein (Figure 4C), suggesting its importance in fetal development and may be closely associated with placental pathology.

Consistent with bioinformatic results, the transcriptional level of Smad2 in the fetal side of the FGR placenta was significantly (*p* < 0.001) lower than that in the healthy placenta (Figure 4E). The stain of Smad2 located in the trophoblast cells was strong in the IHC graph of a fetal side full-term healthy placenta (Figure 4D). Notably, trophoblast cells are better differentiated and developed in healthy placentas, suggesting a vital role of Smad2 in FGR caused by placental pathology. Interestingly, the lower expression of Smad2 was significant (*p* = 0.002 < 0.005, fold change = 0.426) in the fetal side placenta of the FGR group (Figure 4E). Correlation analysis on healthy and FGR subjects significantly demonstrated the negative regulation (r =−0.471, *p* < 1.0 E – 04) of Smad2 and miR-155-5p expression in fetal side placentas (Figure 4F). Thus, miR-155-5p likely blocks the expression of Smad2 by targeting, silencing, or degradation. The downregulated Smad2/miR-155-5p axis may cause the functional disruption of trophoblast cells, contributing to FGR.

### 3.5 Smad2 directly binds to miR-155-5p while Smad2 heterozygote mice presented SGA phenotypes

**FIGURE 5:**
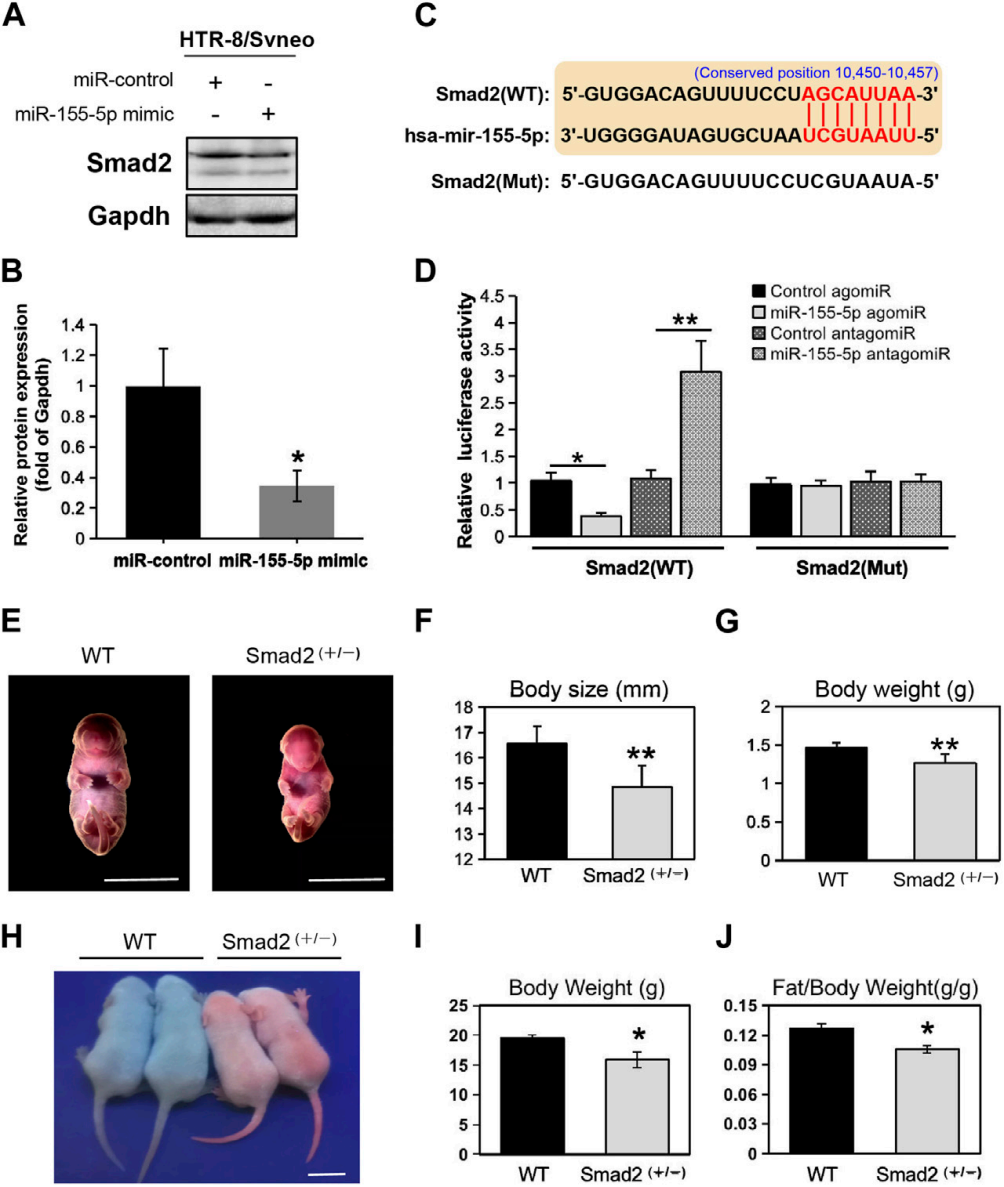
MiR-155-5p directly binds to Smad2 and Smad2 heterozygote mice presented low birth weight. **(A)** Western blot of HTR8/SVneo cells at 48 h post-transfection. **(B)** Grayscale ratio values of Smad2/Gapdh blots presenting in **(A)**. **(C)** The sequence of the mutated site for luciferase reporter assays. **(D)** Smad2 transcript levels in HEK293T cells co-transfected with agomiR, antagomiR and pmir-GLO reporter constructions. **(E)** Smad2^+/−^ heterozygote and WT embryonic mice at E18.5. **(F)** Body sizes of Smad2^+/−^, and WT mice at E18.5 (*n* > 7). **(G)** Body weights of Smad2^+/−^ and WT mice at E18.5 (*n* > 7). **(H)** Photo of Smad2^+/−^ mice and WT littermates (male, at day 10) under fluorescent lamp. Smad2^+/−^ mice presenting red fluorescent. **(I)** Body weights of Smad2^+/−^ and WT mice at 4 weeks (*n* = 7). **(J)** Fat/weight ratio of Smad2^+/−^ and WT mice at 4 weeks (*n* = 7). Scale bars indicate 10 mm *p < 0.05, **p < 0.005.

**FIGURE 6:**
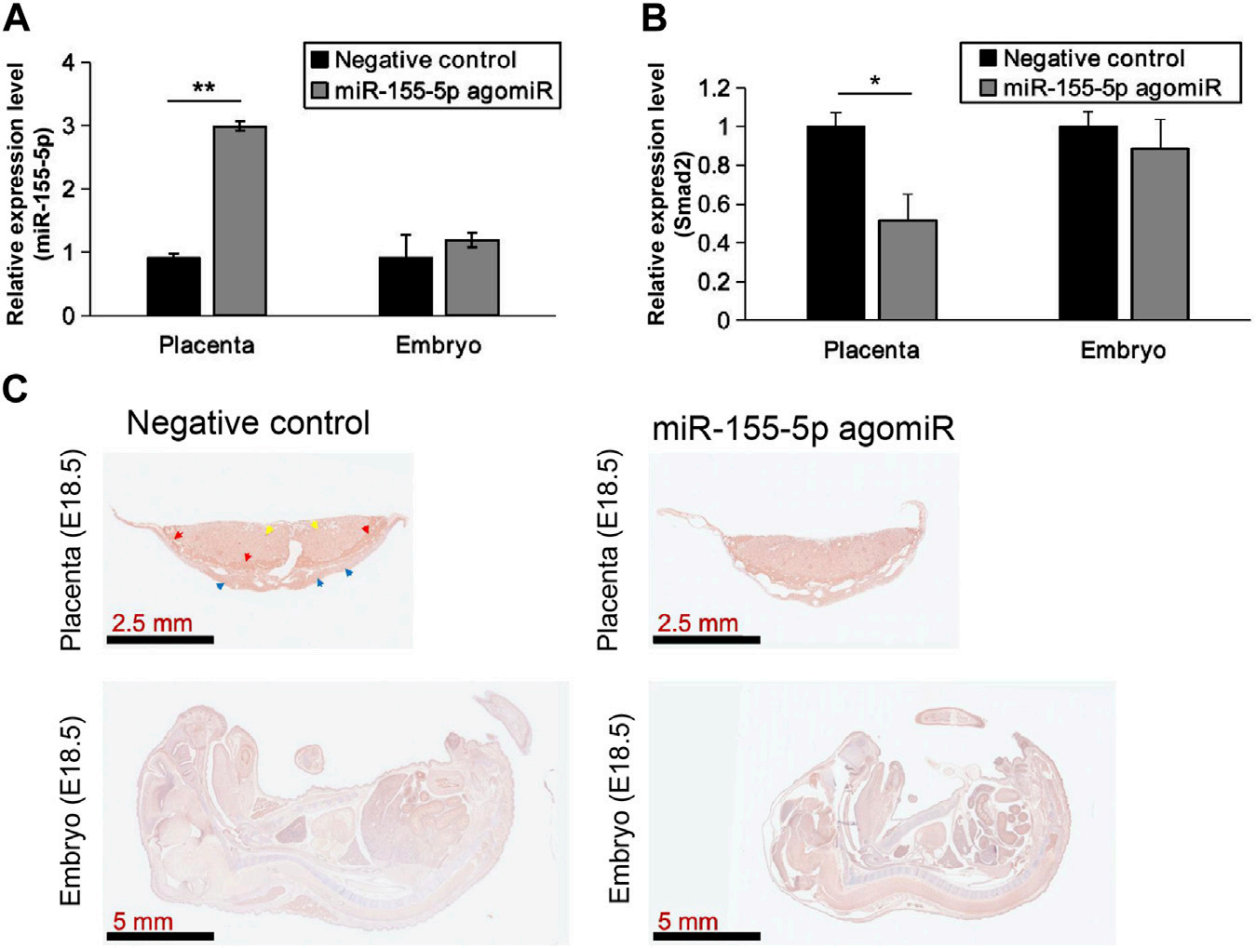
MiR-155-5p agomiR injection changed mice embryo and placentas. **(A)** The transcript level of miR-155-5p in placentas and embryos of mice injected by agomiR (*n* = 3). **(B)** The transcript level of Smad2. **(C)** Smad2 Immunohistochemistry results for placenta tissues and embryos of agomiR injected mice (at E18.5). *p < 0.05, **p < 0.005.

At 48 h post-transfection with the miR-155-5p mimic, the intensity of the bands of Smad2 were shallower (Figures 5A, B). Luciferase reporter plasmids with the WT Smad2 sequence and Mut Smad2 sequence in binding sites of miR-155-5p were generated (Figure 5C). Co-transfection of Smad2 (WT) and miR-155-5p agomiR or antagomiR significantly changed luciferase activity (*p* < 0.05), whereas co-transfection of Smad2 (Mut) did not affect luciferase activity (Figure 5D). These results indicated that Smad2 directly binds to miR-155-5p and acts as a target gene for miR-155-5p. Heterozygous mice were bred to verify the relationship between Smad2 and FGR. Smad2^+/−^ heterozygote mice were smaller and lighter at E18.5, 1 day before birth (Figures 5E–G, *n* > 7). Smad2^+/−^ heterozygote babies (1 week) presenting mCherry fluorescent are smaller than WT littermates (Figure 5H). The body weight and fat composition of baby mice were collected at the fourth week post-natal. Consistently, they were much lighter (*p* = 0.0281 < 0.05, fold change = 0.833) than WT mice in the same litter (*n* > 7) and presented a significantly lower body fat rate (*p* = 0.013 < 0.05, fold change = 0.846) (Figures 5I, J). Furthermore, mice injected with miR-155-5p agomiR presented a much smaller body size (E18.5) and downregulation of Smad2 in the placenta (Figure 6C). The expression level of miR-155-5p (Figure 6A) and Smad2 (Figure 6B) have been determined by qPCR, they have successfully changed by miR-155-5p agomiR in mice placentas.

## 4 Discussion

The placenta grows rapidly during pregnancy and plays a vital role in fetal programming. Insufficient perfusion, nutrient transport defects, and abnormal gas exchange caused by placental injury are common pathologies of FGR (Aplin et al., 2020). MicroRNAs are potential biomarkers and mechanistic features of placentas about pregnancy complications (Sadovsky et al., 2015). Discoveries on the integrative mechanism of miRNA-mRNA help to understand the physiological process of pregnancy, either in a proper or pathophysiological context. In this study, upregulated miR-155-5p was detected in FGR placentas. In total, 128 target genes (PreditedTargets.xls) of miR-155-5p were predicted, defined, and subjected to GO and KEGG pathway enrichment analyses. MiR-155-5p probably negatively regulated the function mentioned by top enriched GO terms of “regulation of biosynthetic process” and “DNA-binding transcription factor activity,” which account for the features of FGR as these GO terms are associated with trophoblast cell proliferation and differentiation (Aplin et al., 1998). Moreover, FGR pathogenesis is associated with malfunction of EVT migration and invasion of uterine spiral arteries. Consistently, the most enriched significant pathway “Notch signaling pathway” is an EVT progenitor marker (Haider et al., 2016). Other enriched pathways like “MAPK signaling” and “HIF-1 signaling” are also well known as regulators for the uterine environment supporting conceptus development (Rajakumar et al., 2007; Lim and Song, 2016). Our results indicated the effects of miR-155-5p on the pathophysiological development of intrauterine FGR environmental homeostasis.

For complete gestation, a balance must be achieved between the proteolytic excavation of the uterine interstitial space to placenta implantation and embryo growth. Specifically, villous cytotrophoblasts divide to produce a multilayered cellular shell and EVT cell columns during early pregnancy. They pass through a transient proliferative period and subsequently exit the cell cycle and differentiate into fully invasive cells (Aplin et al., 1999). EVT cells rapidly invade the superficial termini of the maternal spiral arterioles. Evidences have shown that a subset of giant EVT cells that undergo post-mitotic polyploidization persist until the third trimester in the placental bed, probably as active endocrine agents (al-Lamki et al., 1999; Rai and Cross, 2014). With the onset of cell senescence, those trophoblast-derived giant cells stop migrating and deposit themselves (Vento-Tormo et al., 2018). Corresponding to existing literature, we found that the target gene of Smad2 accumulated in EVT cells of fetal-side placentas in the third trimester (Figures 4D, E), indicating that Smad2 may be essential for EVT cell differentiation and placental homeostasis underlying endocrine regulation. This result probably caused the statistical difference in the qPCR results, as EVT was almost absent in fetal-side FGR placentas. Similarly, the negative association between miR-155-5p and Smad2 was significant in fetal-side placentas (Figure 4F). In addition, miR-155-5p suppressed HTR8 cell proliferation, migration, and invasion, indicating the effects of the Smad2/miR-155-5p axis on the pathophysiology of poor blood perfusion and tissue damage in the placenta of FGR neonates.

As miRNA acts primarily by degrading mRNA transcripts or inhibiting the translation of mRNA, Smad2 expression levels were decreased by the miR-155-5p mimic. Luciferase assays further confirmed the targeting of miR-155-5p on Smad2. Moreover, Smad2^+/−^ heterozygote mice were significantly smaller and lean postnatal mice. The Smad2 protein belongs to the Smad protein family and mediates the signal of transforming growth factor (TGF)-beta, thus regulating multiple cellular processes, such as cell proliferation, apoptosis, and differentiation. Recent research reveals that Bmp2 promoted trophoblast cell invasion by upregulating N-cadherin via non-canonical Smad2 signaling (Zhao et al., 2018), which supports and explains our findings of lower Smad2 levels in FGR placentas. However, it is difficult to clarify the precise mechanism of Smad2/miR-155-5p. The Smad protein mediates multiple signaling pathways, while the placenta is spatially heterogeneous and temporally evolutionary, making the dominant role between Smad2 and miR-155-5p unclear. Identifying signs of impending pathology to intervene and ameliorate disease in later pregnancy remains a complex and challenging objective. Therefore, experiments using cell and animal models focusing on the precise regulation of the physiological and molecular mechanisms of Smad2/miR-155-5p are required and valuable, and may provide a potential therapeutic target during pregnancy.

## Data Availability

The datasets presented in this study can be found in online repositories. The names of the repository/repositories and accession number(s) can be found in the article/Supplementary Materials.

## Supplementary material

SUPPLEMENTARY FIGURE S1 The qPCR analysis of miR-155-5p expression in placental tissues in other literature. *P < 0.05, **P < 0.005.
