# Supplementary figures and images for: An miRNA-mRNA integrative analysis in human placentas and mice: role of the Smad2/miR-155-5p axis in the development of fetal growth restriction
Source: Front Bioeng Biotechnol. 2023 May 18;11:1159805. doi: 10.3389/fbioe.2023.1159805 (PMC10233019; doi:10.3389/fbioe.2023.1159805)

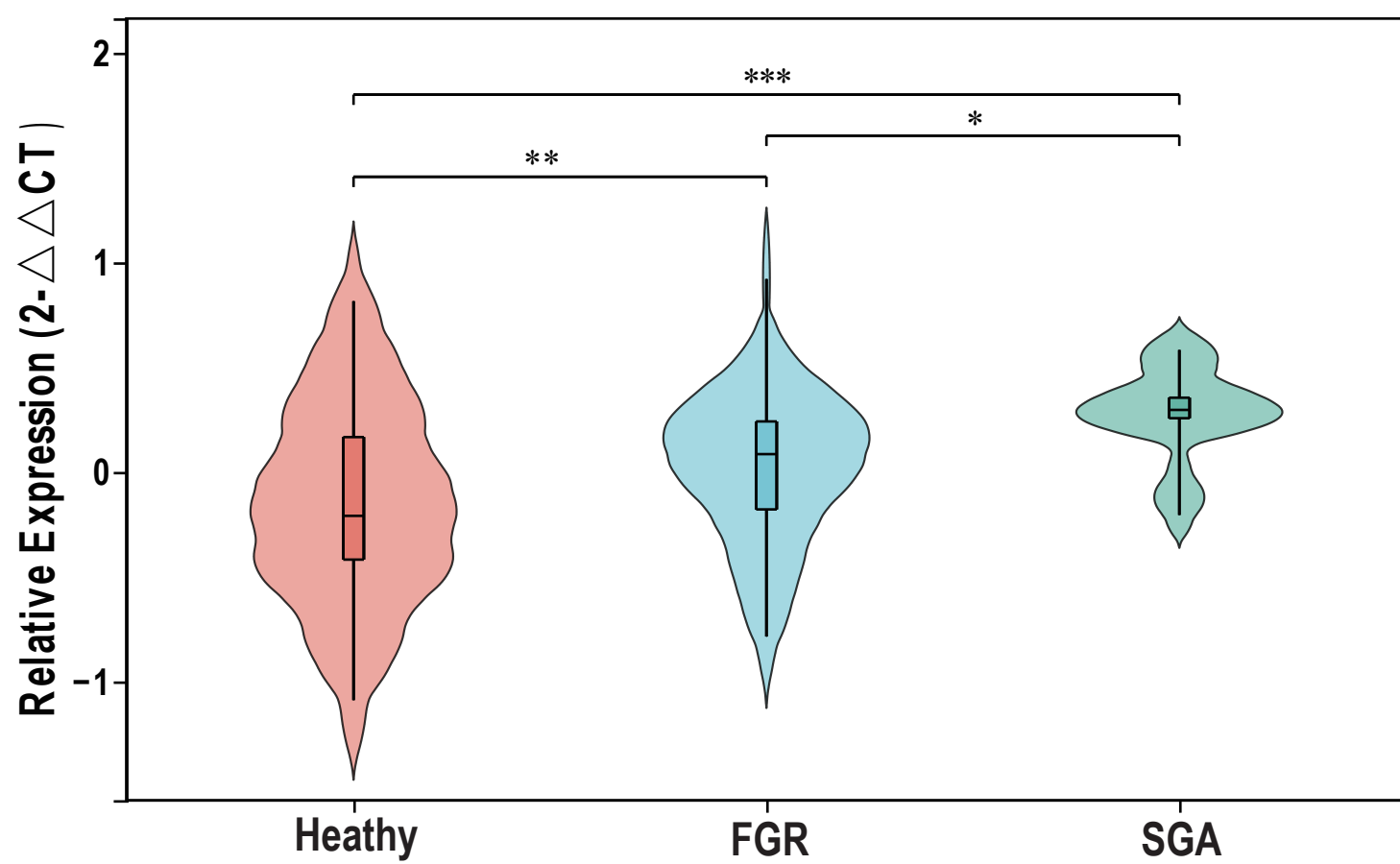

FIGURE S1

The qPCR analysis of miR-155-5p expression in placental tissues in other literature. \*P < 0.05, \*\*P < 0.005.

Supplement: Supplementary file 5 [file Image1.pdf]
